# Patient, clinician, and performance-based measures provide different information about clinical symptoms in patients with severe knee osteoarthritis presenting with depressive symptoms: a cross-sectional study

**DOI:** 10.1186/s12891-023-06971-0

**Published:** 2023-10-23

**Authors:** José Pérez-Maletzki, Fernando Domínguez-Navarro, Sergio Roig-Casasús, Beatriz Díaz-Díaz, Felipe Querol-Giner, Carmen García-Gomáriz, José-María Blasco

**Affiliations:** 1 Group in Physiotherapy of the Ageing Process: Social and Healthcare Strategies, Departament de Fisioteràpia, Universitat de València, Calle Gascó Oliag 5, Valencia, 46010 Spain; 2 Department of Physiotherapy, Faculty of Health Science, Universidad Europea de Valencia, Valencia, Spain; 3 Departament de Fisioterapia, Facultad de Fisioterapia, Universitat de València, València, Spain; 4 Hospital Universitari i Politècnic La Fe de València, València, Spain; 5 Hospital Clínic i Universitari de València, València, Spain; 6 Departament de Podología, Facultad de Podología, Universitat de València, València, Spain

**Keywords:** Outcome measures, Knee osteoarthritis, Depressive symptoms, Patient-reported outcomes, Functional capacity

## Abstract

**Background and purpose:**

Clinical status of subjects with knee osteoarthritis (KOA) is influenced by a complex interaction of several biopsychosocial factors. The use of patient-reported measures (PROM) is considered the gold standard for their evaluation. However, considering that 1 in 5 subjects with KOA present with depressive symptoms, it is necessary to analyse how this psychological domain may influence the subjective perception of PROM. The objective was to study the impact that depressive symptoms have on functional outcome assessments, according to the degree of objectivity of diverse outcome measures.

**Methods:**

Cross-sectional study. Subjects with severe KOA, verified with clinical and radiological symptoms, were assessed with patient-reported (Oxford Knee Score), clinician-reported (knee range of motion), and performance-based (Timed up and go test) measures. The existence of depressive symptoms was assessed with the Yesavage scale, and participants were classified for having no-, mild- or severe-symptoms. Linear correlations (*r*) and one-way analysis of variance compared groups (95% CI).

**Results:**

244 participants were analysed, of which 75 (30.7%) had depressive symptoms. These symptoms had an inverse moderate association with Oxford Knee Score (*r* = -0.387). However, the correlation with the Timed up and go test was low (*r* = 0.176), while there was no correlation with knee ROM (*r* = -0.087).

**Conclusions:**

This study supports that patient-reported questionnaires may offer biased information on the clinical status of patients with severe knee osteoarthritis who present with depressive symptoms. Consideration of such symptoms may be critical to ensure data collected to accurately reflect patients’ capacities and perceptions.

**Supplementary Information:**

The online version contains supplementary material available at 10.1186/s12891-023-06971-0.

## Introduction

Patients with severe knee osteoarthritis (KOA) report persistent pain and limited functional capacity. Both clinical symptoms are influenced by a complex interaction between psychological, pathological, socio-demographic, and disability factors, commonly present in KOA [1–3]. Indeed, these patients frequently present with depressive symptoms associated with their condition [4, 5], with an approximate prevalence of 20% [6–8].

The diagnosis of KOA is verified through radiological evaluation after the appearance of clinical symptoms. A number of psychometrically validated outcome measures are available to assess such symptoms [9]. Regarding pain in KOA, it is considered nociceptive, and patients’ perceptions directly influence their routine assessment with PROM [2, 10, 11]. However, the assessment of functional capacity may depend on whether the point of view is that of the assessor or the patient, and on the degree of objectivity of the outcome measure used: firstly, the so-called clinician-reported measures are claimed to offer objective information (e.g. joint range of motion (ROM), strength); secondly, measures based on performance estimate functional capacity in basic tasks, such as walking or climbing stairs [12]; finally, patient-reported questionnaires are characterized by considering the patient’s perception in a wide range of functional activities, offering a more subjective view [9]. Precisely, these questionnaires have been for several decades the gold standard in KOA [13], and the most frequently used [14].

Previous studies have investigated the association between psychologic status and clinical symptoms in KOA, and how this status can impact on total knee replacement outcome [15]. This verified the existence of a significant association with pain [10, 11, 16]. Regarding functional capacity, some studies appraised such association using PROM specifically. The findings were non-consistent across trials and supported that depressive symptoms could either have an influence on functional assessment [17] or not [18]. Other studies also included performance-based measures to appraise this association [2, 19]. However, no study classified the findings considering the degree of objectivity of the outcome measure used, according to the previously exposed [20, 21].

Ensuring that PROM results accurately reflect clinical symptoms is crucial to successful application. The objective was to evaluate whether the existence of depressive symptoms could influence PROM resultant scores. To this end, the association between depressive symptoms and PROM scores was determined, and then, it was compared whether this association occurred when clinical symptoms were evaluated with outcome measures that provide information from other points of view (i.e., clinician-reported or based on performance).

## Methods

Cross-sectional study recruiting the sample from two university hospitals, Hospital Clínico Universitario and Hospital Universitario y Politécnico La Fe from Valencia, Spain. The University of Valencia was responsible for the integrity and conduct of the study, which adhered to the Helsinki ethical guidelines and successive updates. The procedures were approved by the ethical boards of the institutions (no. 2018/0621 and no. 2018/280). All methods were performed in accordance with the Strengthening the Reporting of Observational Studies in Epidemiology (STROBE) Statement.

All participants were informed verbally and in writing of the purpose of the study and signed an informed consent to participate. Patients aged between 60 and 80 years old, diagnosed with severe KOA, were eligible for the study. The condition was verified with radiography assessment, i.e., a result of 4 on the Kellgren-Lawrence scale (values from 0 to 4), and clinical symptoms, i.e. pain ≥ 3 on the visual analogue scale (VAS, values ​​from 0 to 10). Those participants incapable to follow verbal instructions, or who suffered from some vestibular, neurological and/or cognitive alteration that did not allow the understanding or correct execution of the tests and questionnaires were excluded. The data were collected between May and June 2019.

### Data collection

Potentially eligible participants were referred by two orthopaedic surgeons. Two researchers were in charge of collecting the data and verifying compliance with the eligibility criteria. One of them was assigned to collect demographic and clinical information and explain the tests, while the other supervised their correct execution.

In accordance with the objectives of the study, all patients were assessed for psychological and functional status. Data were collected in a single session.

Psychological status was assessed by determining the presence of depressive symptoms using the Spanish version of the Yesavage Geriatric Depression Scale, a standardised 15-question questionnaire, with yes or no answers, for a final score of 0 to 15, which determines three degrees of severity of depression. This scale has been shown to be a reliable tool to assess depressive symptoms among elderly people, with the Spanish version validated too [22]. The assessment was carried out in an isolated room, where there was only an assessor who verbally explained the instructions of the scale and wrote down the answers offered by the participants.

Functional status was measured in its 3 dimensions with varying degrees of objectivity: patient-, clinician-reported and performance-based. (I) The Spanish version of the Oxford Knee Score (OKS), a patient-reported outcome specifically designed and developed to assess people with severe knee OA, was used as PROM measurement. The Spanish version has been validated, revealing excellent test-retest reliability (ICC 0.993; IC 95%: 0.990–0.995) [23]. The scale consists of 12 questions about an individual’s functional activities of daily living and how they have been affected by typical symptoms of the disease over the past four weeks, reporting from none to maximum impossibility for each activity, resulting in a final score ranging from 0 (minimum functionality) to 48 (maximum functionality). The assessor explained the instructions verbally and the participants completed the questionnaire. (II) Clinician-reported functionality was obtained by measuring the knee range of motion by using a telescopic goniometer, being this measurement validated [24]. (III) Performance-based functionality for common daily tasks, such as walking or getting up from a chair, was estimated with the Timed up and go test (TUG). This test has been shown to describe dynamic balance and mobility, capacities with a relevant impact on functionality [25]. To perform the test, the patient gets up from a chair, walk 3 m, turn around a cone, go back and sit down again in the shortest possible time. Time was measured with a stopwatch, with the shortest time obtained being the highest functionality.

To reinforce analyses and verify the consistency of the results, this study compared the collected data with two factors that had been shown to associate with depressive symptoms: quality of life, estimated with the European Quality of Life-5 Dimensions (EQ-5D) health-related questionnaire [26], and pain, estimated with a visual analogue scale (VAS) with scores that ranged from 0, the worse possible pain, to 10, no pain. The EQ-5D results were calculated according to the database scores available for the Spanish population, with a VAS score from 0, the worse, and 1, the best possible health-related quality of life.

### Analysis of data

A univariate analysis described the clinical and demographic characteristics of the sample, including means, standard deviations and frequencies (SPSS version 22.0, IBM®, licensed from the University of Valencia). The normality of the distribution for the quantitative variables was verified using Kolmogorov-Smirnov test. The possible association between depressive symptoms and function, pain and quality of life was sought. Pearson’s partial correlation coefficients, considered a low, moderate or high association between limits of *r* = 0.2 and 0.5. Furthermore, the sample was classified according to the severity of depressive symptoms, so that scores from < 5, < 10 and up to15, were considered as non-existent, mild or severe depressive symptoms respectively [27]. One-way analysis of variance was performed using the severity of depressive symptoms as an independent factor and the clinical variables (functional capacity, pain, and quality of life) as dependent. All confidence intervals were established at 95%. An estimate of the necessary sample was carried out with *Z*-correlation tests with independent variables in which an effect size *q* = 0.6 was intended, with *α* = 0.05 and *β* = 0.2 [28], for which a sample greater than 150 participants was necessary.

## Results

**Fig. 1:**
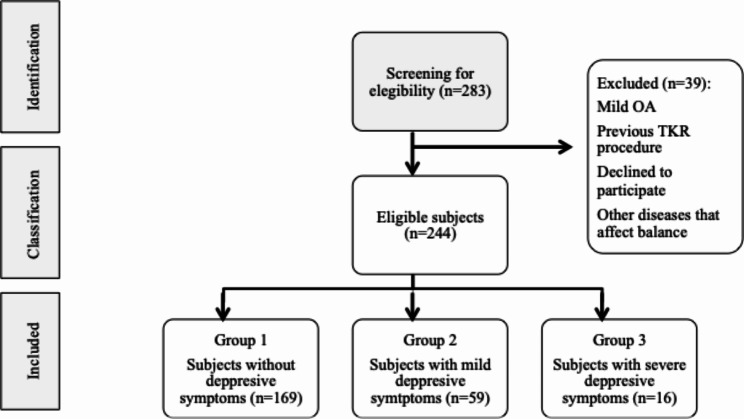
Sample flowchart

Of the 283 potential participants with diagnosed knee OA who were assessed for eligibility, 244 subjects were finally included in the study and analysed. The mean age of the participants was 71.2 (SD 6.9) years, of which 69.3% (n = 169) were women; 75 patients (30.7%) reported mild to severe depressive symptoms (score > 5 Yesavage scale), with a mean response of 4.3 (SD 3.1). The detailed characteristics of the individuals are shown in Table 1 and the diagram of the participants in Fig. 1.

**Table 1:** Sample characteristics

	Mean (SD)
**Characteristics**	
n	244
Women (n, %)	169 (69.3)
Age (y)	71.2 (6.9)
Height (cm)	158.5 (9.5)
Weight (kg)	79.2 (15.5)
Osteoarthritis knee (right/left)	(130/114)
**Clinical symptoms**	
**Functional capacity**	
Patient-reported (Oxford Knee Score)	27.5 (8.5)
Based on performance (Timed up and go)	16.2 (7.4)
Clinician-reported (Knee range of motion)	100.7 (16.9)
**Depression (Yesavage)**	
Overall (score)	4.3 (3.1)
No depression (≤ 5) (n, mean (SD))	169 / 2.6 (1.6)
Mild depression (6–9) (n, mean (SD))	59 / 7.2 (0.9)
Severe depression (≥ 10) (n, mean (SD))	16 / 12.1 (1.3)
**Pain (VAS (0–10))**	5.9 (2.2)
**Quality of life (EQ-5D, VAS (0–1))**	0.5 (0.2)

VAS: Visual Analogue Scale; EQ-5D: EuroQol 5 Dimensions

**Fig. 2:**
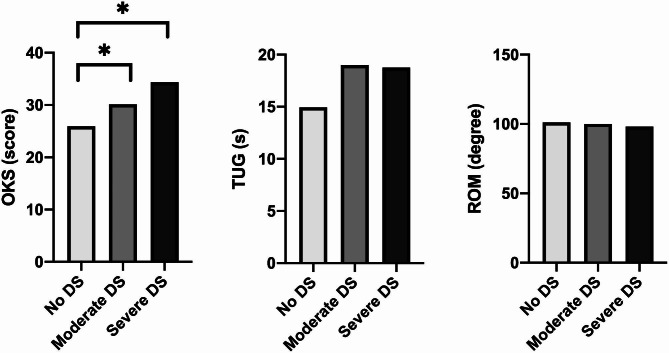
People with no depressive symptoms (DS), moderate DS, or severe DS. Three types of assessments are presented: (1) left side of the figure: patient-reported (OKS: Oxford Knee Score); (2) center, based on performance (TUG: Timed up and go) and (3) right side, clinician-reported (ROM: Knee range of motion). * *p* < 0.001 in one-way ANOVA looking for group (i.e. no, moderate or severe DS) differences. * Indicates between-groups differences with p < 0.01

The data analyses revealed that depressive symptoms did impact self-reported functionality. Specifically, correlation study demonstrated a moderate and inverse relation between depressive symptoms and PROM (Table 2). That implies that as the Yesavage score increases, the OKS score decreases. Furthermore, ANOVA results revealed the significance of symptoms severity on OKS scores (p < 0.001), as illustrated in Fig. 2. However, the correlation coefficient between depressive symptoms and the Timed up and go test was low, while there was no correlation with clinician-reported assessment.

**Table 2:** Association between depression and clinical outcome

	Functional capacity		
	Patient-reported (OKS)	Based on performance (TUG)	Clinician-reported (KROM)
Depression (Yesavage)	-0.387**	-0.176**	-0.087
Pain (VAS)	0.540	0.140	-0.160
Quality of life (EQ-5D)	-0.690	-0.410	0.180

OKS: Oxford Knee Score; TUG: Timed up and go; KROM: Knee range of motion; VAS: Visual Analogue Scale; EQ-5D: EuroQol 5 Dimensions

** Indicates differences with p < 0.01

As expected, pain and quality of life showed a positive association with the Yesavage scale, so that the higher the scores, the lower the quality of life and the greater the pain, as it can be observed in Table 2. Again, there were significant differences according to the severity of the symptoms (*p* < 0.05).

## Discussion

This research found that PROM scores were associated with the existence of depressive symptoms in patients with severe knee OA. However, these symptoms had little or no association with performance-based and clinician-reported functional assessments, respectively. Therefore, this study supports that (1) the existence of depressive symptoms should be evaluated in routine assessments; especially, considering that PROM have been the gold standard for clinical verification in subjects with KOA, and that the rate of depressive symptoms is high (consistently, it was registered an approximate of 30% [7, 8]); (2) functional measures based on both the perception of patients and assessors should be also used in practice. These findings contribute to a more complete picture of the functional status of patients with KOA, and how psychological status influences the patient’s self-perception of health.

It is well established in the current literature that functional status depends on the interaction of various physical and bio-psycho-social factors [16, 29], which has led to a renewed interest over the last decade in understanding how these factors interact with each other and concretely, how depression can impact on a patient’s functional status.^16^ In this context, the results obtained in this study, showing a significant association between depressive symptoms and patient-reported outcomes, are consistent with previous studies [2, 16, 19, 20], although some others failed to demonstrate this association [10, 18]. Therefore, a firm conclusion regarding the existence of an association between depressive symptoms and functional reports cannot yet be drawn, as shown in the review by Calders et al. [21]. In addition, this review also found a large heterogeneity in the sample analyzed, which meant that the overall quality of this evidence was rated as very low. To address this issue, standardization of diagnostic criteria, the adoption of uniform assessment tool, and a large emphasis to identifying and controlling those variables that may interfere with the results (i.e. body mass index, i.e. age) could provide a more comprehensive understanding of the status of patients with KOA and facilitate more robust conclusions [30].

In the present study, while depressive symptoms were significantly correlated with patient-reported outcome, this correlation was low with performance-based measure, and nonexistent with knee ROM. This result supported that depressive symptom may influence the perception that patients with KOA have about their functional capacity, but not necessarily the outcome of less subjective measures or not reliant on patients’ perception. Therefore, when a patient with KOA presents with depressive symptoms, functional outcomes may differ depending on whether objective or self-reported measures are used. Different reasons have been proposed to explain the link between depression and worsening PROMs, as the direction of the cause-effect relationship is not entirely clear, and it is not known what precedes it [31]. It has been suggested that depression contributes to fear-avoidance behavior and kinesiofobia, which may have a negative impact on perceived capacity [32]. On the other hand, patients with KOA who report lower coping skills require more medical attention and are more dissatisfied with the treatment they receive [33]. As poorer functional perception and depression are bidirectionally related and feedback negatively on each other [34], it is first necessary to correctly identify these situations and address them together, providing self-care management programs, in order to break the vicious circle [35].

Objective measurements, such as knee motion or performance-based test (TUG) seems to be less influenced by the depress level of the patient. However, not all the literature seems to agree with this perspective, and some authors did find a significant association between higher depression and worse results of objective functional capacity [2, 16, 35, 36], emphasizing that pain also plays a determining role [2] – consistent with our results. As the literature offers different points of view, it is worth considering the factors that might lead to different results. Some studies analyzed specific populations (i.e. only women [16]) or those with comorbidities (e.g. obese, women [16, 35]). The association between depressive symptoms and functional capacity had already been investigated in patients with KOA, but no study analyzed this relationship by classifying the findings according to the degree of objectivity of the outcome measure. For such reason, it was assessed two additional factors for which the influence of depressive symptoms had been well established: pain and quality of life [10]. As expected, we found a significant association between these factors, which reinforces the findings.

PROMs are considered the gold standard in the functional evaluation of patient with KOA, and the results largely determines the therapeutic procedure to be applied, with those with high levels of pain and disability being eligible to undergo total knee replacement [37]. However, despite their widespread use, PROMs have also faced criticism, due to patient’s reports may be susceptible to influences such as social desirability bias or difficulties in accurately quantifying and recalling pas emotions and experiences [38]. Furthermore, this study suggests that, given the high prevalence of depressive symptoms in patients with KOA, it is important to interpret PROMs results while considering the potential influence of depression, which can confound patients’ perceptions [1].

Regarding clinical application, the findings of this study align with the emerging perspective for comprehending pain in individuals with KOA, transitioning from a purely mechanical viewpoint to a biopsychosocial paradigm [39]. This research contributes new evidence on understanding the influence of psychological state in the patient’s functional status. Within this framework, several actions may be taken. Firstly, a comprehensive assessment of psychological well-being may aid in the recognition of depressive symptoms and the identification of potential barriers affecting patients’ self-assessments of their health. Secondly, the incorporation of objective or self-reported measures can contribute to a more comprehensive and reliable evaluation of the patient, reducing the likelihood of bias. Additionally, this approach may assist in resolving potential discrepancies between radiological and clinical findings [12], ultimately leading to greater specificity when considering personalized or elective treatment options.

As a strength, the sample size and possible external validity should be highlighted, given that the sample was collected in two centers and, in terms of age and sex, it is generalizable to the population suffering from KOA [8].

On the other hand, it is necessary to mention limitations, such as that depressive symptoms and their association with different functional and patient-reported parameters were analyzed, but not with other factors that may be likely related to worse patient-reported functionality, such as the social environment or work of the individual, sex, weight or increasing age. Likewise, in future research it would be interesting to assess the existence and degree of sarcopenia in a population with knee OA, since it is a frequent finding in elderly individuals that is also negatively related to functionality [40]. The prevalence of depressive symptoms in the sample of this study was within the established ranges, although it was slightly higher than the average of other studies (20% vs. 30%) [8], which may influence a more marked decrease in physical activity performed by individuals.

## Conclusion

This study supports that the existence of depressive symptoms associates with worse outcome in patient-reported functional assessments. However, the correlation is very low with functional capacity and non-existent with knee function. Therefore, patient-reported questionnaires may offer a biased perspective of the functional status of patients with severe knee osteoarthritis who present with depressive symptoms. It is recommended to assess the psychological status on a routine basis, and to include functional measures that consider the perspective of clinicians and patients, as this can help in the design of individualized treatments.

### Electronic supplementary material

Below is the link to the electronic supplementary material.

Supplementary Material 1

## Data Availability

The datasets generated or analysed during the current study are available from the corresponding author on reasonable request.
